# Novel Coumarin Derivatives Containing 1,2,4-Triazole, 4,5-Dicyanoimidazole and Purine Moieties: Synthesis and Evaluation of Their Cytostatic Activity 

**DOI:** 10.3390/molecules170911010

**Published:** 2012-09-12

**Authors:** Krešimir Benci, Leo Mandić, Tomislav Suhina, Mirela Sedić, Marko Klobučar, Sandra Kraljević Pavelić, Krešimir Pavelić, Karlo Wittine, Mladen Mintas

**Affiliations:** 1 Department of Organic Chemistry, Faculty of Chemical Engineering and Technology, University of Zagreb, Marulićev trg 19, Zagreb 10000, Croatia; 2 Department of Biotechnology, University of Rijeka, Slavka Krautzeka 83 A, Rijeka 51000, Croatia

**Keywords:** **:** 1,2,4-triazole, 4,5-dicyanoimidazole and purine coumarin derivatives, acyclic nucleoside analogues, antitumor activity evaluation

## Abstract

We report here on the synthesis and *in vitro* anti-tumor effects of a series of novel 1,2,4-triazole (compounds **3**–**6**), 4,5-dicyanoimidazole (compound **7**), and purine (compounds **8**–**13**) coumarin derivatives and their acyclic nucleoside analogues **14**–**18**. Structures of novel compounds **3**–**18** were deduced from their ^1^H- and ^13^C-NMR and corresponding mass spectra. Results of anti-proliferative assays performed on a panel of selected human tumor cell lines revealed that compound **6** had moderate cytostatic activity against the HeLa cell line (IC_50_ = 35 µM), whereas compound **10** showed moderate activity against the HeLa (IC_50_ = 33 µM), HepG2 (IC_50_ = 25 µM) and SW620 (IC_50_ = 35 µM) cell lines. These compounds showed no cytotoxic effects on normal (diploid) human fibroblasts.

## 1. Introduction

Coumarin (1,2-benzopyrone or 2*H*-1-benzopyran-2-one) and its derivatives are ubiquitously distributed in Nature and many of them exhibit diverse and useful biological activities [1,2]. These compounds have numerous medical applications including antitumor and anti-HIV therapy [3,4], central nervous system (CNS) stimulation [5], antibacterial [6,7], anti-inflammatory [8,9,10] and anti-coagulant properties [11]. In addition, hydroxycoumarins are known to be powerful chain-breaking anti-oxidants which can prevent free radical injury by scavenging reactive oxygen species [12,13]. Some coumarin derivatives display cytostatic properties, while others have cytotoxic activities [14]. For example, coumarin and its active metabolite, 7-hydroxycoumarin, have demonstrated growth-inhibitory activity in human cancer cell lines, such as A549 (lung), ACHN (renal), H727 (lung), MCF-7 (breast) and HL-60 (leukemia), and have also been reported to have anti-proliferative activity in prostate cancer, malignant melanoma and metastatic renal cell carcinoma in clinical trials [15,16,17,18]. The recent discovery of coumarins having weak estrogenic activity resulted in the use of such derivatives as therapeutic agents in preventing the emergence of menopause-related diseases, such as osteoporosis, increased risk of cardiovascular disease and cognitive deficiencies [19]. Furthermore, the substituted benzopyranobenzothiazinones exhibited estrogenic activity in MCF-7 breast carcinoma cells [20]. Of particular interest in breast cancer chemotherapy is the finding that some coumarin analogs and their active 7-hydroxycoumarin metabolites have sulfatase and aromatase inhibitory activities. Coumarin-based selective estrogen receptor modulators (SERMs) and coumarin estrogen conjugates have also been described as potential anti-breast cancer agents. Since breast cancer is the second leading cause of death in American women after lung cancer, there is a strong impetus to identify potential new drug treatments for breast cancer [21]. 

The anti-tumor activities of coumarin and its known metabolite 7-hydroxycoumarin were tested in several human tumor cell lines by Steffen *et al.* [22]. Both compounds inhibited cell proliferation of gastric carcinoma cell line (HSC-39), colon carcinoma cell line (Caco-2), hepatoma-derived cell line (Hep-G2) and lymphoblastic cell line (CCRF). Egan *et al.* [23] have synthesized, characterized and determined cytostatic and cytotoxic nature of 8-nitro-7-hydroxycoumarin using both human (including K-562 and HL-60) and animal cell lines grown *in vitro*. The effect of warfarin on tumor cell growth was studied [24]. Warfarin inhibits metastasis of Mtln3 rat mammary carcinoma without affecting primary tumor growth. Seven known coumarins showing significant cytotoxic activities on P388 cell lines were isolated from the roots of *Angelica gigas* (Umbelliferae) [25]. The cytotoxicity of 22 natural and semi-synthetic simple coumarins was evaluated in human small cell lungcarcinoma cell line GLC4 and human colorectal cancer cell line COLO 320 using the MTT assay [26]. Furthermore, a number of 4-hydroxycoumarin derivatives have been studied for their HIV integrase inhibitory potency [27]. The main purpose was to simplify the large structure of the compounds while maintaining their potency. It was found that the minimum active pharmacophore consisted of coumarin dimer containing aryl substituent on the central linker, methylene. Additionally, 1,2,4-triazole represents a unique template that is associated with anti-viral, anti-bacterial, anti-fungal, anti-inflammatory and CNS activity. Compounds incorporating 1,2,4-triazole rings have also been shown to be anti-tumor agents [28]. Pyrimidine, 1,2,4-triazole and purine derivatives are constituents of a number of useful drugs and are associated with many biological, pharmaceutical and therapeutic activities. Condensed pyrimidine, 1,2,4-triazole and purine derivatives have been reported as anti-microbial, analgesic, anti-viral, anti-inflammatory, anti-HIV, anti-tubercular, anti-tumor, anti-malarial, diuretic and cardiovascular [29,30,31,32] agents.

**Figure 1:**
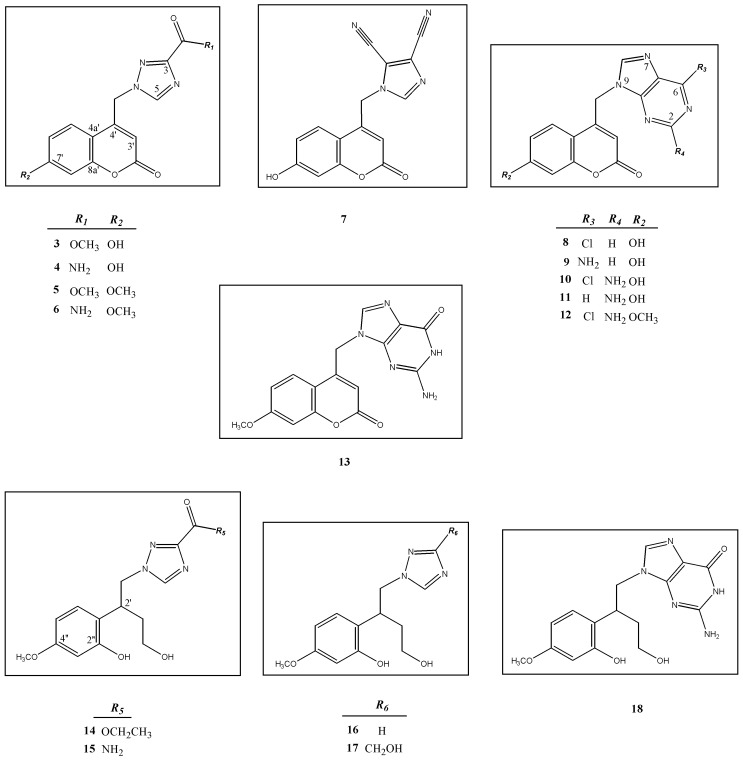
New coumarin derivatives containing 1,2,4-triazole (**3**–**6**), 4,5-dicyanoimidazole (**7**) and purine (**8**–**13**) moiety, their open ring analogues (**14**–**17**) as well as acyclic nucleoside analogue (**18**).

In light of these findings and based on our previous study [33], we efficiently synthesized a series of new 7-methoxy- or 7-hydroxycoumarin derivatives containing 1,2,4-triazole-3-carboxylic methyl ester (**3** and**5**) or 3-carboxyamide moieties as heterocyclic constituents of ribavirin (compounds **4** and **6**), 4,5-dicyanoimidazole (compound **7**) or substituted purine derivatives (compounds **8**–**13**), their open ring analogues (compounds **14**–**17**) and acyclic nucleoside analogue (compound **18**) (Figure 1).

## 2. Results and Discussion

### 2.1. Chemistry

**Scheme 1:**
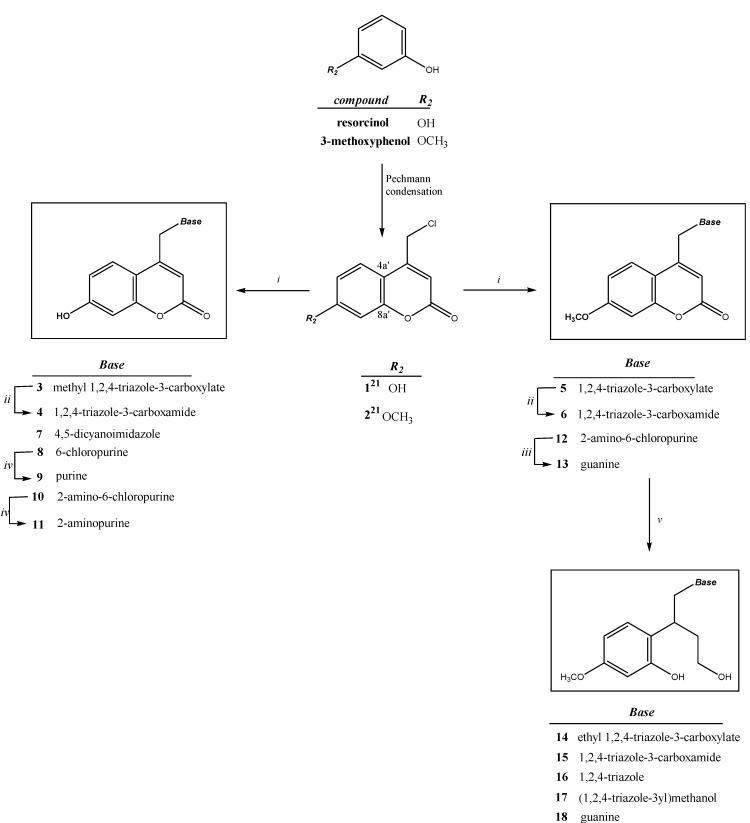
Synthesis of new coumarin derivatives containing 1,2,4-triazole (**3**–**6**), 4,5-dicyanoimidazole (**7**) and purine (**8**–**13**) moiety, their open ring analogues (**14**–**17**) as well as acyclic nucleoside analogue (**18**).

The syntheses of new structurally diverse 7'-methoxy- or 7'-hydroxycoumarin derivatives containing 1,2,4-triazole (compounds **3**–**6**), 4,5-dicyanoimidazole (compound **7**) and purine (compounds **8**–**13**) moieties, their open ring analogues (compounds **14**–**17**) and acyclic nucleoside analogue (compound **18**) were carried out by the sequence of reactions shown in Scheme 1. These syntheses were performed by coupling of the synthetic precursors 4-(chloromethyl)-7-hydroxy-2*H*-chromen-2-one (**1**) or 4-(chloromethyl)-7-methoxy-2*H*-chromen-2-one (**2**) with appropriate heterocyclic bases. The synthesis of 4-chloromethylcoumarins **1** and **2** involving the Pechmann condensation was performed starting from resorcinol and 3-methoxyphenol, respectively, according to the pathway shown in Scheme 1 and as described previously [23]. 

*Reagents and conditions*: (i) DMF, NaH, 80 °C, nucleoside bases; (ii) gaseous NH_3_, MeOH, room temperature; (iii) 80% HCO_2_H, 100 °C; (iv) 80% HCO_2_H, 100 °C, then 29% aq. NH_3_, rt; (v) NaBH_4_, EtOH (dry), 70 °C.

Subsequent reduction of **5** and **6** with NaBH_4_ gave the corresponding open ring analogues **14**–**17**, whereas reduction of **13** with NaBH_4_ gave the acyclic nucleoside analogue **18**. During the reduction of the starting methyl ester **5** in ethanol solution with NaBH_4_ three products have been isolated, namely compounds **14**, **16** and **17**. Trans-esterification occured producing ethyl ester derivative **14**. The methyl ester group of compound **5** was removed giving the open ring analogue **16**. Reduction of methyl ester group of compound **5** occurred also giving open ring analogue **17** containing 1,2,4-triazole-yl-3-methanol moiety. 

### 2.2. NMR Assignments

The structures of the newly synthesized compounds were deduced from the analysis of their ^1^H- and ^13^C-NMR and mass spectra. The assignment of ^1^H-NMR spectra was performed on the basis of the chemical shifts, substituent induced chemical shifts, signal intensities, magnitude and multiplicity of H-H coupling constants. The chemical shifts in ^1^H and ^13^C-NMR spectra (Table 1, Table 2 and Experimental) are in concordance with the proposed structures of the novel compounds and related coumarine derivatives [23].

### 2.3. Antiproliferative Effects

Compounds **3**–**18** were evaluated for their inhibitory activities against human tumor cell lines: HeLa (cervical carcinoma), MCF-7 (breast epithelial adenocarcinoma, metastatic), HepG2 (hepatocellular carcinoma), SW620 (colorectal adenocarcinoma, metastatic), as well as on normal (diploid) human fibroblasts (control cell line BJ) (Table 3). Table 2,Table 1. Of all evaluated compounds, only compound **6** containing a 1,2,4-triazole-3-carboxyamide moiety, the heterocyclic constituent of the ribavirin, showed moderate cytostatic activity against HeLa cells (IC_50_ = 35 µM), whereas compound **10** involving a modified guanine base showed moderate activity against HeLa (IC_50_ = 33 µM), HepG2 (IC_50_ = 25 µM) and SW620 (IC_50_ = 35 µM) cells. 

**Table 1:** ^1^H-NMR (DMSO-d_6_) chemical shifts (δ/ppm) and H-H coupling constants (*J*/Hz) in ^1^H-NMR spectra for compounds **3**–**13** (for enumeration of atoms *c.f.* Figure 1).

	OH-7'	H-5	H-8	H-5'	H-6'	H-8'	NH_2_-2	CH_2_-N	H-3'	OMe-7'
3 ^a^	10.72 (s, 1H)	8.34 (s, 1H)	/	7.72 (d, 1H, *J* = 8.8)	6.87 (AB, dd, 1H, *J* = 2.3, 8.7)	6.78 (d, 1H, *J* = 2.2)	/	6.01 (s, 2H)	5.30 (s, 1H)	/
4 ^b^	10.72 (s, 1H)	8.25 (s, 1H)	/	7.74 (d, 1H, *J* = 8.7)	6.85 (AB, dd, 1H, *J* = 2.0, 8.6)	6.77 (d, 1H, *J* = 2.0)	/	6.06 (s, 2H)	5.26 (s, 1H)	/
5 ^c^	/	8.09 (s, 1H)	/	7.77 (d, 1H, *J* = 8.9)	7.05 (AB, dd, 1H, *J* = 2.5, 8.6)	7.01 (AB, dd, 1H, *J* = 2.6, 8.8)	/	5.84 (s, 2H)	5.76 (s, 1H)	3.85 (s, 3H)
6 ^d^	/	8.83 (s, 1H)	/	7.73 (d, 1H, *J* = 8.9)	7.07 (AB, dd, 1H, *J* = 2.0, 8.6)	7.02 (AB, dd, 1H, *J* = 2.0, 8.8)	/	5.81 (s, 2H)	5.79 (s, 1H)	3.87 (s, 3H)
7	10.76 (s, 1H)	8.45 (s, 1H)	/	7.66 (d, 1H, *J* = 8.7)	6.87 (AB, dd, 1H, *J* = 2.2, 8.7)	6.80 (d, 1H, *J* = 2.1)	/	5.82 (s, 2H)	5.76 (s, 1H)	/
8 ^d^	10.72 (s, 1H)	/	8.82 (s, 1H)	7.80 (d, 1H, *J* = 8.7)	6.87 (AB, dd, 1H, *J* = 2.0, 8.6)	6.78 (d, 1H, *J* = 2.1)	/	5.81 (s, 2H)	5.61 (s, 1H)	/
9 ^f^	10.72 (s, 1H)	/	8.10 (s, 1H)	7.77 (d, 1H, *J* = 8.7)	6.89 (AB, dd, 1H, *J* = 2.3, 8.7)	6.81 (d, 1H, *J* = 2.2)	/	5.65 (s, 2H)	5.36 (s, 1H)	/
10	10.72 (s, 1H)	/	8.22 (s, 1H)	7.79 (d, 1H, *J* = 8.7)	6.87 (AB, dd, 1H, *J* = 2.2, 8.7)	6.78 (d, 1H, *J* = 2.2)	6.76 (s, 2H)	5.55 (s, 2H)	5.44 (s, 1H)	/
11 ^g^	10.01 (s, 1H)	/	7.78 (s, 1H)	7.74 (d, 1H, *J* = 8.8)	6.84 (AB, dd, 1H, *J* = 1.9, 8.7)	6.76 (d, 1H, *J* = 2.2)	6.75 (s, 2H)	5.42 (s, 2H)	5.23 (s, 1H)	/
12	/	/	8.20 (s, 1H)	7.85 (d, 1H, *J* = 8.8)	7.01 (AB, dd, 1H, *J* = 2.0, 8.7)	7.05 (d, 1H, *J* = 1.9)	6.97 (s, 2H)	5.56 (s, 2H)	5.54 (s, 1H)	3.87 (s, 3H)
13 ^h^	/	/	7.78 (s, 1H)	7.86 (d, 1H, *J* = 8.8)	7.02 (AB, dd, 1H, *J* = 1.9, 8.7)	7.06 (d, 1H, *J* = 1.9)	6.53 (s, 2H)	5.46 (s, 2H)	5.41 (s, 1H)	3.88 (s, 3H)

^a^ Compound **3**: signal for COOCH_3_-triazole: 3.88 ppm (s, 3H); ^b^ Compound **4**: signal for CONH_2_-triazole: 8.38 and 8.07 ppm (2 × s, 2 × 1H); ^c^ Compound **5**: signal for COOCH_3_-triazole: 3.87 ppm (s, 3H); ^d^ Compound **6**: signal for CONH_2_-triazole: 7.84 and 7.64 ppm (2 × s, 2 × 1H); ^e^ Compound **8**: signal for H-2-purine: 8.80 ppm (s, 1H); ^f^ Compound **9**: signal for NH_2_-6-purine: 7.97 ppm (s, 2H); H-2-purine: 8.10 ppm (s, 1H);^ g^ Compound **11**: signal for H-6-purine: 8.45 ppm (s, 1H); ^h^ Compound **13**: signal for NH-purine: 10.67 ppm (s, 1H).

**Table 2:** ^1^H-NMR (DMSO-d_6_) chemical shifts (δ/ppm) and H-H coupling constants (*J*/Hz) in ^1^H-NMR spectra for compounds **14–18** (for enumeration of atoms *c.f.* Figure 1).

	OH-2"	H-8	H-5	H-6"	H-5"	H-3"	OH-4'	OMe-4"	H-1'	H-2'	H-3'	H-4'
14 ^a^	9.64 (s, 1H)	/	8.11 (s, 1H)	6.88 (AB, dd, 1H, *J*= 3.27, 8.43)	6.36 (AB, dd, 1H, *J* = 2.34, 8.36)	6.36 (d, 1H, *J* = 8.30)	5.19 (t, 1H, *J* = 6.03)	3.65 (s, 3H)	3.17–3.24 (m, 2H)	2.52–2.56 (m, 1H)	1.80–1.61 (m, 2H)	4.15–4.18 (m, 2H)
15 ^b^	9.49 (s, 1H)	/	8.25 (s, 1H)	6.91 (d, 1H, *J* = 8.37)	6.35 (d, 1H, *J* = 2.31)	6.30 (AB, dd, 1H, *J*= 2.28, 8.34)	5.11 (t, 1H, *J* = 6.01)	3.65 (s, 3H)	3.16–3.18 (m, 2H)	2.32–2.35 (m, 1H)	1.79–1.61 (m, 2H)	4.37–4.40 (m, 2H)
16 ^c^	9.47 (s, 1H)	/	8.17 (s, 1H)	6.87 (d, 1H, *J* = 8.40)	6.34 (d, 1H, *J* = 2.28)	6.28 (AB, dd, 1H, *J* = 2.28, 8.40)	5.14 (t, 1H, *J* = 5.96)	3.65 (s, 3H)	3.25–3.17 (m, 2H)	2.84–2.87 (m, 1H)	1.79–1.61 (m, 2H)	4.27–4.29 (m, 2H)
17 ^d^	9.42 (s, 1H)	/	8.07 (s, 1H)	6.91 (d, 1H, *J* = 8.40)	6.36 (d, 1H, *J* = 2.46)	6.31 (AB, dd, 1H, *J* = 2.40, 8.34)	5.11 (t, 1H, *J* = 6.06)	3.66 (s, 3H)	3.21–3.13 (m, 2H)	2.33–2.37 (m, 1H)	1.79–1.61 (m, 2H)	4.34–4.37 (m, 2H)
18 ^e^	9.46 (s, 1H)	7.2 (s, 1H)	/	6.92 (d, 1H, *J* = 8.34)	6.32 (d, 1H, *J* = 1.95)	6.29 (AB, dd, 1H, *J* = 2.35, 8.19)	4.97 (t, 1H, *J* = 6.04)	3.66 (s, 3H)	3.26–3.17 (m, 2H)	3.04–3.07 (m, 1H)	1.82–1.62 (m, 2H)	4.13– 4.16 (m, 2H)

^a^ Compound **14**: signal for COOCH_2_CH_3_-triazole: 2.70 ppm (m, 2H); COOCH_2_CH_3_-triazole: 1.05 ppm (t, 3H,*J* = 7.1 Hz); ^b^ Compound **15**: signal for CONH_2_-triazole: 7.69 and 7.50 ppm (2 × s, 2 × 1H); ^c^ Compound **16**: signal for H-3: 7.88 ppm (s, 1H); ^d^ Compound **17**: signal for OH-3-triazole: 4.37 ppm (d, 1H,*J* = 6.0 Hz); CH_2_-3-triazole: 4.30 (d, 2H, *J* = 7.4 Hz); ^e^ Compound **18**: signal for NH-purine: 10.70 ppm (s, 1H); NH_2_-purine: 6.56 ppm (s, 2H).

These compounds showed no cytotoxic effects in normal fibroblasts. It appears that compounds **6** and **10** exert their effects in a different way that is probably attributable to a different genetic background of tumour cell lines. Compound **6** having a 1,2,4-triazole-3-carboxamide ligand is highly selective towards human cervix cancer (HeLa) cells. These cells harbor integrated Human Papillomavirus (HPV) genomes and express two *viral oncogenes*, E6 and E7, which inactivate the p53and pRB tumor suppressors. Compound **10** with a 2-amino-6-chloropurine ligand exerted noticeable cytostatic effect on other tumour cell lines as well, more precisely on hepatic carcinoma (HepG2) and colon cancer (SW620) cells both bearing mutations in the *p53* gene probably through impairment od DNA synthesis. Similarly, literature data on 1,2,4-triazolylcoumarins indicate potential antitumor and anti-HIV activities of this class of compounds [34]. Similarly to compound 10 with chloropurine moiety, literature data report on chloropurine derivatives with cytostatic activity towards many cancer cell lines including colon cancer [35].

**Table 3:** Inhibitory effects of compounds **3**–**18** on the growth of malignant tumor cell lines in comparison with their effects on the growth of normal diploid fibroblasts (BJ). The results are presented as IC_50_ values (μM).

IC_50_^a^ (μM)					
Substance No.	Cell lines				
	HeLa	MCF-7	HepG2	SW620	BJ
3	>100	>100	>100	>100	>100
4	>100	>100	>100	>100	>100
5	>100	>100	>100	>100	>100
6	35.5 ± 13.5	>100	>100	>100	>100
7	>100	>100	>100	>100	>100
8	>100	>100	>100	>100	>100
9	>100	>100	>100	>100	>100
10	34 ± 8.4	>100	25.6 ± 12.6	35.4 ± 3.7	>100
11	>100	>100	>100	>100	>100
12	>100	>100	>100	>100	>100
13	>100	>100	>100	>100	>100
14	>100	>100	>100	>100	>100
15	>100	>100	>100	>100	>100
16	>100	>100	>100	>100	>100
17	>100	>100	>100	>100	>100
18	>100	>100	>100	>100	>100

^a^ IC_50_ represents the concentration of a drug that is required for 50% growth inhibition *in vitro*.

## 3. Experimental

### 3.1. General

The melting points (uncorrected) were determined with a Kofler micro hot-stage (Reichert, Vienna, Austria). Pre-coated Merck silica gel 60F-254 plates were used for thin layer chromatography (TLC) and the spots were detected under UV light (254 nm). Column chromatography (CLC) was performed using silica gel (0.063–0.2 mm, Sigma-Aldrich, Co., 3050 Spruce Street, St. Luis, MO 63103 USA); glass column was slurry-packed under gravity. Mass spectra were recorded on an Agilent 6410 instrument (Agilent Technoligies, Wilmington, NC, USA) equipped with electrospray interface and triple quadrupole analyzer (LC/MS/MS). High-performance liquid chromatography was performed on Agilent 1100 series system with UV detection (photodiode array detector) using Zorbax C18 reverse-phase analytical column (2.1 × 30 mm, 3.5 µm, Agilent). Structures of newly synthesized compounds were deduced on the basis of analysis of their ^1^H and ^13^C-NMR as well as their mass spectra and confirmed by elemental analysis. ^1^H and ^13^C-NMR spectra were acquired on a Bruker 300 MHz NMR spectrometer (Bruker Spectrospin, Rheinstetten, Germany). All data were recorded in solvent DMSO-*d*_6_ at 298 K and chemical shifts are referred to TMS. Individual resonances were assigned on the basis of their chemical shifts, signal intensities, multiplicity of resonances and H-H coupling constants. Elemental analyses were performed in the Central Analytic Service, Rudjer Bošković Institute Zagreb, Croatia, using a Perkin Elmer 2400 Elemental Analyser.

### 3.2. Procedures for the Preparation of Compounds

#### 3.2.1. Compounds **3–13**

Compounds **3**–**13** were prepared by the following general procedure: to a stirred solution containing methyl 1*H*-1,2,4-triazole-3-carboxylate, 1*H*-1,2,4-triazole, 1*H*-4,5-dicyanoimidazole, 2-amino-6-chloro-1*H*-purine or 6-chloro-1*H*-purine and NaH (1.5 equiv.) in DMF (30 mL), either 4-chloromethyl-7-hydroxycoumarin (**1**, 500 mg, 2.37 mmol) (for the preparation of compounds **3** and **4** and **7**–**11**) or 4-chloromethyl-7-methoxycoumarin (**2**, 500 mg, 2.23 mmol) (for the preparation of compounds **4** and **5**, **12** and **13**) was added after 2h at room temperature under moisture-free conditions. The reaction mixture was stirred at 80 °C overnight, evaporated and the residual crude oil purified by column chromatography (CH_2_Cl_2_–MeOH = 50:1) to give pure compounds **3**–**13** as white solids.

#### 3.2.2. Compound Data

*Methyl 1-((7-hydroxy-2-oxo-2H-chromen-4-yl)methyl)-1,2,4-triazole-3-carboxylate* (**3**).This compound was synthesized following the general procedure (Section 3.2.1) using methyl *1H*-1,2,4-triazole-3-carboxylate (302 mg, 2.37 mmol) to give **3** (310 mg, 44%, m.p. = 216–218 °C). ^13^C-NMR (DMSO) δ/ppm: 162.1 (C-2a'), 160.3 (C=O-triazole), 158.2 (C-7a'), 155.4 (C-3-triazole), 151.3 (C-9a'), 145.4 (C-4a'), 140.5 (C-5-triazole), 126.4 (C-5a'), 113.7 (C-3a'), 109.9 (C-10a'), 108.4 (C-6a'), 103.0 (C-8a'), 53.5 (COOCH_3_-triazole), 50.8 (CH_2_-N); MS *m/z* 302.1 [M+1]. Elemental analysis. Calc. for C_14_H_11_N_3_O_5_: C 55.82, H 3.68, N 13.95, Found: C 55.96, H 3.74, N 13.97.

*1-((7-Hydroxy-2-oxo-2H-chromen-4-yl)methyl)-1,2,4-triazole-3-carboxamide* (**4**). Methyl 1-((7-hydroxy-2-*oxo*-2*H*-chromen-4-yl)methyl)-1,2,4-triazole-3-carboxylate (**3**, 200 mg, 0.66 mmol) was treated with NH_3_ (g) in MeOH (15 mL) solution at 0 °C in ice bath for 30 min. The resulting mixture was stirred at room temperature overnight. It was evaporated and the residual white solid was separated by column chromatography (CH_2_Cl_2_–MeOH = 40:1) to give white solids of **4** (151 mg, 79%, m.p. = 246–248 °C). ^13^C-NMR (DMSO) δ/ppm: 162.2 (C-2a'), 160.4 (C=O-triazole), 159.0 (C-7a'), 155.4 (C-3-triazole), 151.9 (C-9a'), 147.6 (C-4a'), 141.3 (C-5-triazole), 126.4 (C-5a'), 113.7 (C-3a'), 109.9 (C-10a'), 108.5 (C-6a'), 103.0 (C-8a'), 50.4 (CH_2_-N); MS *m/z* 287.1 [M+1]. Elemental analysis. Calc. for C_13_H_10_N_4_O_4_: C 54.55, H 3.52, N 19.57, Found: C 54.59, H 3.56, N 19.53.

*Methyl 1-((7-Methoxy-2-oxo-2H-chromen-4-yl)methyl)-1,2,4-triazole-3-carboxylate* (**5**). The compound was synthesized following the general procedure (Section 3.2.1) using methyl *1H*-1,2,4-triazole-3-carboxylate (283 mg, 2.23 mmol) to give **5** (528 mg, 75%, m.p. = 234–237 °C). ^13^C-NMR (DMSO) δ/ppm: 162.8 (C-2a'), 159.7 (C=O-triazole), 160.8 (C-7a'), 155.1 (C-3-triazole), 150.6 (C-9a'), 149.6 (C-4a'), 141.7 (C-5-triazole), 125.8 (C-5a'), 112.4 (C-3a'), 110.6 (C-10a'), 110.3 (C-6a'), 101.2 (C-8a'), 56.0 (O-CH_3_), 52.2 (COOCH_3_-triazole), 49.2 (CH_2_-N); MS *m/z* 316.1 [M+1]. Elemental analysis. Calc. for C_16_H_15_N_3_O_5_: C 58.36, H 4.59, N 12.76, Found: C 58.42, H 4.61, N 12.81. 

*1-((7-Methoxy-2-oxo-2H-chromen-4-yl)methyl)-1,2,4-triazole-3-carboxamide* (**6**). Methyl 1-((7-methoxy-2-oxo-2*H*-chromen-4-yl)methyl)-1,2,4-triazole-3-carboxylate (**5**, 500 mg, 1.59 mmol) was treated with NH_3_ (g) in MeOH (20 mL) solution at 0 °C in ice bath for 30 min. The resulting mixture was stirred at room temperature overnight. It was evaporated and the residual white solid was separated by column chromatography (CH_2_Cl_2_–MeOH = 40:1) to give white solids of **6** (402 mg, 84%, m.p. = 261–264 °C). ^13^C-NMR (DMSO) δ/ppm: 163.2 (C-2a'), 160.8 (C=O-triazole), 160.2 (C-7a'), 155.5 (C-3-triazole), 150.4 (C-9a'), 149.3 (C-4a'), 141.3 (C-5-triazole), 126.3 (C-5a'), 112.9 (C-3a'), 111.2 (C-10a'), 111.0 (C-6a'), 101.6 (C-8a'), 56.4 (O-CH_3_), 56.4 (CH_2_-N); MS *m/z* 301.1 [M+1]. Elemental analysis. Calc. for C_14_H_12_N_4_O_4_: C 56.00, H 4.03, N 18.66, Found: C 56.06, H 4.07, N 18.59.

*1-((7-Hydroxy-2-oxo-2H-chromen-4-yl)methyl)-4,5-dicyanoimidazole* (**7**). The compound was synthesized following the general procedure (Section 3.2.1) using 1*H*-4,5-dicyanoimidazole (280 mg, 2.37 mmol) to give **7** (489 mg, 71%, m.p. = 238–240 °C). ^13^C-NMR (DMSO) δ/ppm: 162.3 (C-2a'), 160.2 (C-7a'), 149.4 (C-9a'), 144.5 (C-4a'), 132.6 (C-5-imidazole), 126.3 (C-5a'), 122.7 and 122.9 (2 × C≡N-imidazole), 113.8 (C-3a'), 112.8 and 112.9 [(C-2+C-3)-imidazole)], 109.4 (C-10a'), 108.9 (C-6a'), 103.1 (C-8a'), 47.3 (CH_2_-N); MS *m/z* 293.1 [M+1]. Elemental analysis. Calc. for C_15_H_8_N_4_O_3_: C 61.65, H 2.76, N 19.17, Found: C 61.61, H 2.79, N 19.09.

*1-((7-Hydroxy-2-oxo-2H-chromen-4-yl)methyl)-6-chloropurine* (**8**). The compound was synthesized following the general procedure (Section 3.2.1) using 1*H*-6-chloropurine (367 mg, 2.37 mmol) to give **8** (512 mg, 66%, m.p. > 300 °C). ^13^C-NMR (DMSO) δ/ppm: 162.1 (C-2a'), 160.3 (C-7a'), 155.5 (C-9a'), 152.5 (C-4-purine), 152.4 (C-2-purine), 150.6 (C-6-purine), 149.9 (C-4a'), 143.6 (C-8-purine), 132.3 (C-5-purine), 126.4 (C-5a'), 113.7 (C-3a'), 109.8 (C-10a'), 109.2 (C-6a'), 103.1 (C-8a'), 44.0 (CH_2_-N); MS *m/z* 329.1 [M+1]. Elemental analysis. Calc. for C_15_H_9_ClN_4_O_3_: C 54.81, H 2.76, N 17.04, Found: C 54.76, H 2.72, N 17.08.

*1-((7-Hydroxy-2-oxo-2H-chromen-4-yl)methyl)purine* (**9**). Compound **8** (300 mg, 0.87 mmol) was heated in 85% aq formic acid (20 mL) at 100 °C for 3 h. Next, the mixture was evaporated and without further purification suspended in 90% aq EtOH (15 mL) and treated with 29% ammonia for 1h at room temperature, evaporated again and the residual solid was separated by column chromatography (CH_2_Cl_2_–MeOH = 30:1) to give **9** as white solid (197 mg, 73%, m.p. = 275–277 °C). ^13^C-NMR (DMSO) δ/ppm: 162.6 (C-2a'), 160.4 (C-7a'), 157.1 (C-9a'), 155.9 (C-4-purine), 151.8 (C-2-purine), 149.0 (C-4a'), 148.2 (C-6-purine), 145.1 (C-8-purine), 135.2 (C-5-purine), 126.2 (C-5a'), 113.9 (C-3a'), 109.6 (C-10a'), 108.3 (C-6a'), 103.1 (C-8a'), 43.7 (CH_2_-N); MS *m/z* 295.1 [M+1]. Elemental analysis. Calc. for C_15_H_10_N_4_O_3_: C 61.22, H 3.43, N 19.04, Found: C 61.13, H 3.39, N 19.07.

*1-((7-Hydroxy-2-oxo-2H-chromen-4-yl)methyl)-2-amino-6-chloropurine* (**10**). The compound was synthesized following the general procedure (Section 3.2.1) using 1*H*-2-amino-6-chloropurine (402 mg, 2.73 mmol) to give **10** (512 mg, 55%, m.p. > 300 °C). ^13^C-NMR (DMSO) δ/ppm: 162.1 (C-2a'), 160.5 (C-7a'), 160.3 (C-9a'), 154.6 (C-4-purine), 151.3 (C-2-purine), 150.9 (C-6-purine), 150.3 (C-4a'), 142.9 (C-8-purine), 126.3 (C-5a'), 125.9 (C-5-purine), 113.8 (C-3a'), 109.8 (C-10a'), 108.3 (C-6a'), 103.1 (C-8a'), 43.4 (CH_2_-N); MS *m/z* 343,72 [M+1]. Elemental analysis. Calc. for C_15_H_10_ClN_5_O_3_: C 52.41, H 2.93, N 20.37, Found: C 52.47, H 2.97, N 20.31.

*1-((7-Hydroxy-2-oxo-2H-chromen-4-yl)methyl)-2-aminopurine* (**11**). Compound **10** (400 mg, 1.16 mmol) was heated in 85% aq. formic acid (20 mL) at 100 °C for 3 h. Next, the mixture was evaporated and without further purification suspended in 90% aq. EtOH (15 mL) and treated with 29% ammonia for 1h at room temperature. The solution was evaporated and the residual solid was separated by column chromatography (CH_2_Cl_2_–MeOH = 40:1) to give **11** as white solid (197 mg, 52%, m.p. > 300 °C). ^13^C-NMR (DMSO) δ/ppm: 163.5 (C-2a'), 160.6 (C-7a'), 157.4 (C-9a'), 155.6 (C-4-purine), 150.9 (C-2-purine), 149.3 (C-6-purine), 152.4 (C-4a'), 153.4 (C-8-purine), 126.1 (C-5a'), 126.0 (C-5-purine), 114.2 (C-3a'), 109.1 (C-10a'), 107.2 (C-6a'), 103.1 (C-8a'), 43.1 (CH_2_-N); MS *m/z* 309,28 [M+1]. Elemental analysis. Calc. for C_15_H_11_N_5_O_3_: C 58.25, H 3.58, N 22.64, Found: C 58.18, H 3.54, N 22.69.

*1-((7-Methoxy-2-oxo-2H-chromen-4-yl)methyl)-2-amino-6-chloropurine* (**12**). The compound was synthesized following the general procedure (Section 3.2.1) using 1*H*-2amino-6-chloropurine (378 mg, 2.23 mmol) to give **12** (328 mg, 41%, m.p. > 300 °C). ^13^C-NMR (DMSO) δ/ppm: 162.8 (C-2a'), 160.0 (C-7a'), 159.7 (C-9a'), 155.0 (C-4-purine), 150.6 (C-2-purine), 150.1 (C-6-purine), 149.8 (C-4a'), 151.9 (C-8-purine), 125.6 (C-5a'), 125.4 (C-5-purine), 112.5 (C-3a'), 109.4 (C-10a'), 109.0 (C-6a'), 101.1 (C-8a'), 56.0 (O-CH_3_), 42.9 (CH_2_-N); MS *m/z* 357,75 [M+1]. Elemental analysis. Calc. for C_16_H_12_ClN_5_O_3_: C 53.72, H 3.38 N 19.58, Found: C 53.76, H 3.42, N 19.61.

*1-((7-Methoxy-2-oxo-2H-chromen-4-yl)methyl)guanine* (**13**).Compound **12** (500 mg, 1.40 mmol) was heated in 85% aq formic acid (20 mL) at 100 °C for 3 h. Next, the mixture was evaporated and the residual solid was separated by column chromatography (CH_2_Cl_2_–MeOH = 30:1) to give **13** as white solid (197 mg, 52%, m.p. > 300 °C). ^13^C-NMR (DMSO) δ/ppm: 162.8 (C-2a'), 160.9 (C-7a'), 159.8 (C-9a'), 156.9 (C-6-purine), 156.7 (C-4-purine), 154.0 (C-2-purine), 151.5 (C-4a'), 151.3 (C-8-purine), 125.6 (C-5a'), 121.9 (C-5-purine), 112.5 (C-3a'), 110.5 (C-10a'), 108.6 (C-6a'), 101.1 (C-8a'), 56.0 (O-CH_3_), 42.7 (CH_2_-N); MS *m/z* 340.1 [M+1]. Elemental analysis. Calc. for C_16_H_13_N_5_O_4_: C 56.64, H 3.86, N 20.64, Found: C 56.59, H 3.82, N 20.67.

#### 3.2.3. Compounds **14–18**

Compounds 14–18 were prepared according to the following general procedure: compounds **5**, **6** and **12**, **13** were treated with NaBH_4_ (3 equiv.) in EtOH (20 mL) at 70 °C for 5 h. The reaction mixture were evaporated and the residual oils were separated by column chromatography (CH_2_Cl_2_–MeOH = 10:1) to give colorless oils of the corresponding 1,2,4-triazole (compound **14**–**17**) and guanine (compound **18**) derivatives.

#### 3.2.4. Compound Data

*Ethyl 1-(4-hydroxy-2-(2-hydroxy-4-methoxyphenyl)butyl)-1,2,4-triazole-3-carboxylate* (**14**). *1-(4-hydroxy-2-(2-hydroxy-4-methoxyphenyl)butyl)-1,2,4-triazole* (**16**) and *ethyl 1-(4-hydroxy-2-(2-hydroxy-4-methoxyphenyl)butyl)-1,2,4-triazole-3-hydroxymethyl* (**17**). The compounds were synthesized following the general procedure (Section 3.2.3) using 1-((7-methoxy-2-oxo-2H-chromen-4-yl)methyl)-1,2,4-triazole-3-carboxylate (**5**, 1,000 mg, 3.17 mmol) to give **14** (289 mg, 31%), **16** (256 mg, 28%) and **17** (153 mg, 17%).

*Compound* **14**: ^13^C-NMR (DMSO) δ/ppm: 171.7 (C=O-triazole), 163.9 (C-3-triazole), 159.5 (C-4"), 156.5 (C-2"), 145.2 (C-5-triazole), 129.7 (C-6"), 119.6 (C-1"), 104.7 (C-5"), 101.8 (C-3"), 66.3 (CH_2_-triazole), 59.1 (C-4'), 57.3 (C-1'), 55.2 (O-CH_3_), 53.0 (C-3'), 35.0 (C-2'), 16.7 (CH_3_-triazole); MS *m/z* 336.2 [M+1]. Elemental analysis. Calc. for C_16_H_21_N_3_O_5_: C 57.30, H 6.31, N 12.53, Found: C 57.21, H 6.27, N 12.45.

*Compound* **16**: ^13^C-NMR (DMSO) δ/ppm: 158.6 (C-4"), 156.1 (C-2"), 151.6 (C-3-triazole), 144.1 (C-5-triazole), 129.2 (C-6"), 119.1 (C-1"), 104.2 (C-5"), 101.3 (C-3"), 59.9 (C-4'), 58.7 (C-1'), 54.7 (O–CH_3_), 52.5 (C-3'), 34.5 (C-2'); MS *m/z* 264.1 [M+1]. Elemental analysis. Calc. for C_13_H_17_N_3_O_3_: C 59.30, H 6.51, N 15.96, Found: C 59.36, H 6.46, N 15.91.

*Compound* **17**: ^13^C-NMR (DMSO) δ/ppm: 149.2 (C-3-triazole), 156.6 (C-4"), 156.0 (C-2"), 144.7 (C-5-triazole), 129.7 (C-6"), 119.3 (C-1"), 104.8 (C-5"), 101.8 (C-3"), 59.3 (C-4'), 59.2 (C-1'), 57.3 (CH_2_-triazole), 55.2 (O-CH_3_), 53.7 (C-3'), 35.0 (C-2'); MS *m/z* 294.1 [M+1]. Elemental analysis. Calc. for C_14_H_19_N_3_O: C 57.33, H 6.53, N 14.33, Found: 57.27, H 6.49, N 14.29.

*1-(4-Hydroxy-2-(2-hydroxy-4-methoxyphenyl)butyl)-1,2,4-triazole-3-carboxamide* (**15**). This compound was synthesized following the general procedure (Section 3.2.3) using 1-((7-methoxy-2-oxo-2*H*-chromen-4-yl)methyl)-1,2,4-triazole-3-carboxamide **6** (1500 mg, 5.0 mmol) to give **15** (118 mg, 5.5%). ^13^C-NMR (DMSO) δ/ppm: 163.9 (C=O-triazole), 161.1 (C-3-triazole), 159.5 (C-4"), 156.1 (C-2"), 145.1 (C-5-triazole), 129.1 (C-6"), 119.3 (C-1"), 104.2 (C-5"), 101.4 (C-3"), 59.1 (C-4'), 61.4 (C-1'), 60.2 (O-CH_3_), 53.0 (C-3'), 35.0 (C-2'); MS *m/z* 307.1 [M+1]. Elemental analysis. Calc. for C_14_H_18_N_4_O_4_: C 54.89, H 5.92, N 18.29, Found: C 54.82, H 5.87, N 18.22.

*1-(4-Hydroxy-2-(2-hydroxy-4-methoxyphenyl)butyl)guanine* (**18**). This compound was synthesized following the general procedure (Section 3.2.3) using 1-((7-methoxy-2-oxo-2*H*-chromen-4-yl)methyl)-guanine (**13**, 483 mg, 1.42 mmol) to give **18** (11 mg, 2.2%). ^13^C-NMR (DMSO) δ/ppm: 169.2 (C-5-purine), 160.3 (C-6-purine), 159.1 (C-2-purine), 157.2 (C-4"), 156.8 (C-2"), 153.9 (C-4-purine), 142.7 (C-8-purine), 129.8 (C-6"), 119.7 (C-1"), 104.7 (C-5"), 101.8 (C-3"), 60.2 (C-4'), 59.3 (C-1'), 55.2 (O-CH_3_), 47.2 (C-3'), 35.3 (C-2'); MS *m/z* 346.1 [M+1]. Elemental analysis. Calc. for C_16_H_19_N_5_O_4_: C 55.64, H 5.55, N 20.28, Found: C 55.58, H 5.53, N 20.31.

#### 3.2.5. Cytostatic Activity Assay

This study was set out to examine the anti-proliferative effects of coumarin derivates (**3**–**18**) on human tumor cell lines and normal (diploid) human fibroblasts. 

Cell culturing: Human cell lines HeLa (cervical carcinoma), SW620 (colorectal adenocarcinoma, metastatic), MCF-7 (breast epithelial adenocarcinoma, metastatic), HepG2 (hepatocellular carcinoma) and BJ (normal diploid human fibroblasts) were cultured as monolayers and maintained in Dulbecco’s modified Eagle medium (DMEM) supplemented with 10% fetal bovine serum (FBS), 2 mM L-glutamine, 100 U/mL penicillin and 100 μg/mL streptomycin in a humidified atmosphere with 5% CO_2_ at 37 °C.

Proliferation assay: The cell lines were inoculated into a series of standard 96-well microtiter plates on day 0 at seeding density of 3,000–6,000 cells per well depending upon their specific doubling times. Freshly prepared dilutions of test compounds in culture medium in the concentration range 1 × 10^−8^–1 × 10^−4^ M were added to the microtiter plates, and the cells were grown for further 72 h. The solvent (DMSO) was also tested for its potential inhibitory activity by adjusting its concentration to the values used for the preparation of the working concentrations (DMSO concentration never exceeded 0.1%). After 72 h of incubation, the cell growth rate was evaluated by performing the MTT assay [36]: experimentally determined absorbance values were transformed into the cell percentage growth (PG) using the formulas proposed by NIH and described previously [37]. This method directly relies on control cells behaving normally at the day of assay commencement because it compares the growth of treated cells with that of untreated cells in control wells on the same plate. The results are therefore a percentile difference from the calculated expected value. The IC_50_ value for each compound was calculated from dose-response curves using linear regression analysis by fitting the mean test concentrations that give PG values above and below the reference value. If, however, all of the tested concentrations produce PGs exceeding the respective reference level of effect (e.g., PG value of 50) for a given cell line, the highest tested concentration is assigned as the default value (in the screening data report that default value is preceded by a “>” sign). Each test point was performed in quadruplicate in three individual experiments. The results were statistically analyzed (ANOVA, Tukey post-hoc test at *p* < 0.05).

## 4. Conclusions

A series of novel coumarin derivatives containing heterocyclic bases 1,2,4-triazole (compounds **3**–**6**), 4,5-dicyanoimidazole (compound **7**) and purine (compounds **8**–**13**) linked *via* methylenic spacer to 7'-methoxy- or 7'-hydroxycoumarin moieties were synthesized and evaluated for their potential cytostatic activity. The biological evaluation of these coumarin derivatives generally showed very weak antiproliferative effects for all tested compounds and no cytotoxicity on normal human fibroblasts. Only two compounds (compounds **6** and **10**) exerted stronger effects at higher tested concentrations. This effect was non-specific and completely absent in the micromolar range. It appears that compounds **6** and **10** exert their effects in a different way that is probably attributable to a different genetic background of the tumour cell lines. Compound **6** having a 1,2,4-triazole-3-carboxamide ligand is highly selective towards human cervix cancer (HeLa) cells. These cells harbour integrated Human Papillomavirus (HPV) genomes and express two viral oncogenes, E6 and E7, which inactivate the p53 and pRB tumour suppressors. Compound **10** with a 2-amino-6-chloropurine ligand exerted noticeable cytostatic effect on other tumour cell lines as well, more precisely on hepatic carcinoma (HepG2) and colon cancer (SW620) cells both bearing mutations in the p53 gene probably through impairment of DNA synthesis. Similarly, literature data on 1,2,4-triazolylcoumarins indicate potential antitumor and anti-HIV activities of this class of compounds [34]. Like compound **10** with a chloropurine moiety, the literature data report on chloropurine derivatives with cytostatic activity towards many cancer cell lines including colon cancer [35].
